# The Calm Before the Storm: A Pheochromocytoma Case Report

**DOI:** 10.7759/cureus.46915

**Published:** 2023-10-12

**Authors:** Sally Trinh, Gloria Coronel-Couto

**Affiliations:** 1 Internal Medicine, Florida International University, Herbert Wertheim College of Medicine, Miami, USA

**Keywords:** catecholamines, adrenal tumor, adrenal mass, adrenal lesion, adrenal glands, adrenal incidentalomas, nausea, pheochromocytoma crisis, hypokalemia, pheochromocytoma

## Abstract

Pheochromocytomas are rare tumors that arise from the sympathetic ganglia or adrenal medulla and secrete catecholamines that are known for the classic triad of headaches, profuse sweating, and paroxysmal hypertension. However, there have been instances of asymptomatic patients found to have a pheochromocytoma. Adrenal incidentalomas are accidentally discovered through radiologic imaging, and subsequent testing can confirm a pheochromocytoma. Here, we present a case of a 67-year-old female found to have an adrenal incidentaloma on kidney ultrasound (US) after presenting to the emergency room (ER) due to nausea. She had minimally elevated creatinine at the ER. At her follow-up with her primary care provider (PCP), a kidney US was ordered and showed a probable right suprarenal mass. Further abdominal computed tomography (CT) and abdominal magnetic resonance imaging (MRI) showed that the mass was indeed adrenal, but its etiology is considered indeterminant. Although asymptomatic, further biochemical tests showed elevated serum and urine metanephrines and normetanephrines. Together, these findings revealed that the adrenal mass was likely a silent pheochromocytoma. She underwent a successful right adrenalectomy with pathology confirming pheochromocytoma. This case adds to the literature on the existence of silent pheochromocytomas and highlights the importance of following up on any abnormal findings with a PCP. This patient, although asymptomatic from her pheochromocytoma, needed surgery to prevent possible pheochromocytoma crises, which could cause cardiovascular complications and even death.

## Introduction

Pheochromocytomas are rare catecholamine-secreting tumors that arise from chromaffin cells from either the sympathetic ganglia or adrenal medulla. The release of catecholamines, such as epinephrine, is what causes the classic triad of headaches, profuse sweating, and sustained or paroxysmal hypertension seen in patients with pheochromocytomas [1]. Other less common symptoms seen in patients with pheochromocytoma include orthostatic hypotension, hyperglycemia, insulin resistance, and leukocytosis [2]. However, some patients remain asymptomatic. The diagnosis of pheochromocytomas may be established through tests, such as elevated 24-hour urinary fractionated metanephrines or elevated plasma fractionated metanephrines, and these tests have been used for decades [3]. The diagnosis of pheochromocytomas is usually made first with a biochemical confirmation of catecholamine hypersecretion and then followed by imaging studies to identify the tumor location [4]. At times, an adrenal mass is found in radiographic studies done for other symptoms or reasons. The prevalence of catecholamine-producing pheochromocytomas or extra adrenal chromaffin cells is about 0.1% in those with hypertension and 4% in those incidentally discovered adrenal masses [5]. There have been described instances of asymptomatic patients that were discovered to have a pheochromocytoma incidentally. In these cases, the terms “silent,” “non-functioning,” and “non-secretory” have been used to describe patients that have absence of symptoms [6,7]. These have been included in the category of an adrenal incidentaloma, which is defined as a mass lesion >1 cm in diameter that is accidentally discovered through radiologic imaging [8]. Overall, it is important to pursue systematic workup when a patient is found to have an adrenal incidentaloma. If pheochromocytomas are left untreated, there is a risk of continued hypertension or new onset severe hypertension, which can lead to cardiovascular complications, such as heart failure or myocardial infarction, and possible death [9].

## Case presentation

A 67-year-old female with a past medical history of hypertension, high cholesterol, prediabetes, hyperlipidemia, degenerative joint disease, and questionable transient ischemic attack (TIA) presented to the emergency room (ER) due to nausea that started in the morning when she got up. She denied vomiting or abdominal pain. Since the nausea persisted, she sought care at the ER. She had never had anything similar to this before. Her chronic hypertension was well-controlled, and her questionable TIA was being followed by a neurologist. She had occasional, sporadic episodes of “weird” feelings in her right hand lasting for three seconds. She had been feeling well otherwise, given all her chronic conditions. She denied any flushing, diaphoresis, or tachycardia. The patient willingly consented to this case report so that the medical profession can learn about her silent pheochromocytoma. Table 1 shows her at-home medications.

**Table 1:** List of the patient's at-home medications mg: milligrams; μg: micrograms

Medication	Dose	Frequency
Clopidogrel	75 mg	Daily
Gabapentin	300 mg	Three times a day
Amlodipine	5 mg	Daily
Atorvastatin	10 mg	Daily
Atenolol	25 mg	½ tablet daily
Metformin XR	750 mg	Daily
Hydrochlorothiazide	12.5 mg	Daily
Cetirizine	10 mg	Daily
Vitamin B12	250 μg	Daily
Biotin	5 mg	Daily

The general appearance shows an obese female in no acute distress. The vital signs are as follows: blood pressure 134/79 mm/hg, pulse 66 beats per minute, temperature 98°F, respiratory rate 14 breaths per minute, and BMI 33 kg/m^2^. Cardiac, pulmonary, and abdominal physical exams were unremarkable.

**Figure 1:**
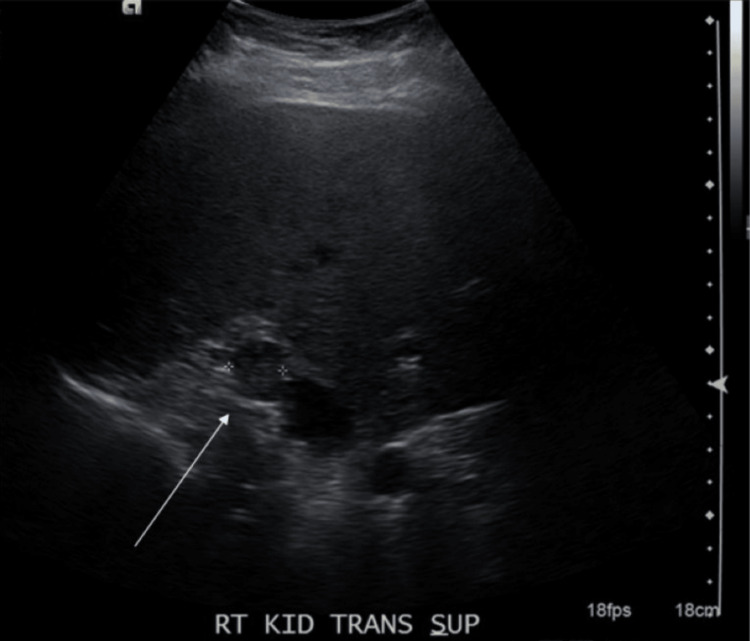
Kidney US displaying a questionable right kidney mass US: ultrasound

Initial laboratory workup revealed hypokalemia of 2.6 mmol/L, elevated non-fasting glucose at 152 mg/dL, and creatinine of 1.1 mg/dL, which increased from the patient’s baseline of 0.93 mg/dL (Table 2). At that time, the ER discontinued the hydrochlorothiazide due to the hypokalemia, and she was discharged from the ER without any knowledge of abnormal labs. She was told to follow up with her PCP. A kidney US was ordered by her PCP due to her elevated creatinine from baseline, which revealed a questionable right kidney or suprarenal mass of 1.9 x 1.9 x 1.6 cm (Figure 1). Both kidneys showed minimally increased echogenicity.

**Table 2:** Emergency room visit lab results mmol/L: millimoles per liter; mg/dL: milligrams per deciliter

Blood work test	Test results	Reference range	Interpretation
Sodium	145 mmol/L	135-145 mmol/L	Normal
Potassium	2.6 mmol/L	3.7-5.2 mmol/L	Decreased
Non-fasting glucose	152 mg/dL	<140 mg/dL	Elevated
Creatinine	1.1 mg/dL	0.6-1 mg/dL	Elevated

**Figure 2:**
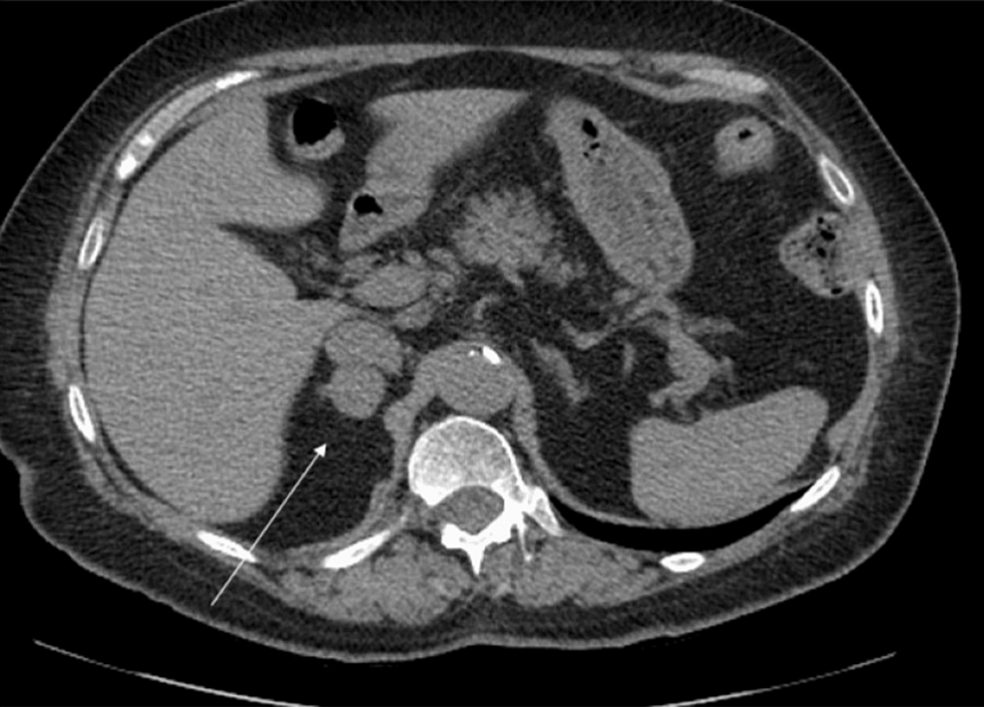
CT abdomen with a hypodense lesion in the right adrenal gland CT: computed tomography

The abnormal findings on kidney US prompted further evaluation, and an abdominal CT scan was ordered. The imaging identified a left adrenal mass consistent with fat density and a mass in the right adrenal gland of indeterminant density (Figure 2). Lab workup revealed elevated normetanephrine and metanephrines on a 24-hour urine collection of 1631 μg/g creatinine and 229 μg/g creatinine, respectively (Table 3). A low-dose cortisol suppression test was performed with the administration of dexamethasone at night, which resulted in a morning cortisol level within normal limits of 1.3 μg/dL that ruled down Cushing's syndrome. Further labs showed a normal aldosterone/renin level of 2.42 ng/dL, and hyperaldosteronism was ruled down. Repeat labs continued to show an increase in urine normetanephrine and metanephrine levels of 1450 μg/g creatinine and 176 μg/g creatinine, respectively (Table 4). Total metanephrines and free plasma normetanephrines were also elevated accordingly at 1626 μg/g creatinine and 534 μg/g creatinine. The patient was then advised to see an endocrinologist due to concerns of possible pheochromocytoma. 

**Table 3:** Initial laboratory data after the adrenal mass was confirmed on CT scan CT: computed tomography; µg/g: micrograms per gram creatinine; ng/dL: nanograms per deciliter; ng/mL/hr: nanograms per milliliter per hour; µg/dL: micrograms per deciliter

24-hour urine collection	Test results	Reference range	Interpretation
Normetanephrines	1631 µg/g creatinine	108-524 µg/g creatinine	Elevated
Metanephrines	229 µg/g creatinine	21-153 µg/g creatinine	Elevated
Aldosterone	6.8 ng/dL	<30 ng/dL	Normal
Renin activity	0.28 ng/mL/hr	0.2-3.3 ng/mL/hr	Normal
Aldosterone/renin ratio	2.42 ng/dL	0.9-28.9 ng/dL	Normal
Cortisol	1.3 µg/dL	10-20 µg/dL	Decreased

**Table 4:** Repeat confirmatory labs done with the PCP PCP: primary care physician; µg/g creatinine: microgram per gram creatinine; pg/mL: picogram per milliliter

Urine and serum test	Test results	Reference range	Interpretation
Urine metanephrines	176 µg/g creatinine	21-153 µg/g creatinine	Elevated
Urine normetanephrines	1450 µg/g creatinine	108-524 µg/g creatinine	Elevated
Total metanephrines	1626 µg/g creatinine	149-603 µg/g creatinine	Elevated
Free metanephrines	36 pg/mL	<57 pg/mL	Normal
Plasma-free metanephrines	534 pg/mL	<148 pg/mL	Elevated

**Figure 3:**
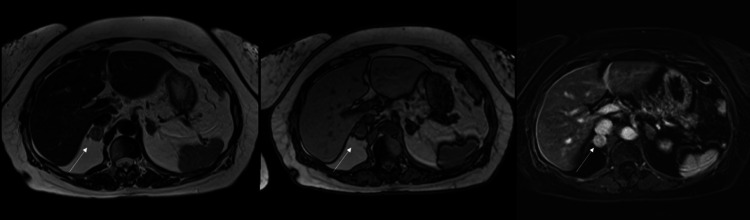
MRI abdomen showing lesions in the right adrenal gland in T2, out-of-phase T1, and T1 C+ (Gd) MRI: magnetic resonance imaging; Gd: gadolinium

During the patient’s endocrinologist visits, she denied any striae, bruising, dizziness, diaphoresis, tachycardia, or swelling. An abdominal MRI further showed a 1.8 cm left adrenal adenoma and 2.2 cm right indeterminate adrenal nodule (Figure 3). A meta-iodobenzylguanidine (MIBG) scan displayed intense MIBG uptake corresponding to the 2.2 cm indeterminate adrenal nodule on the right that suggested an underlying pheochromocytoma. At this time, her beta blocker was stopped, and the patient was referred to a surgeon who recommended right laparoscopic adrenalectomy with initiation of prazosin for alpha-adrenergic blockade. During this time, the patient asked if she could travel via airplane to attend a reunion but was advised not to due to concerns of a pheochromocytoma crisis.

**Figure 4:**
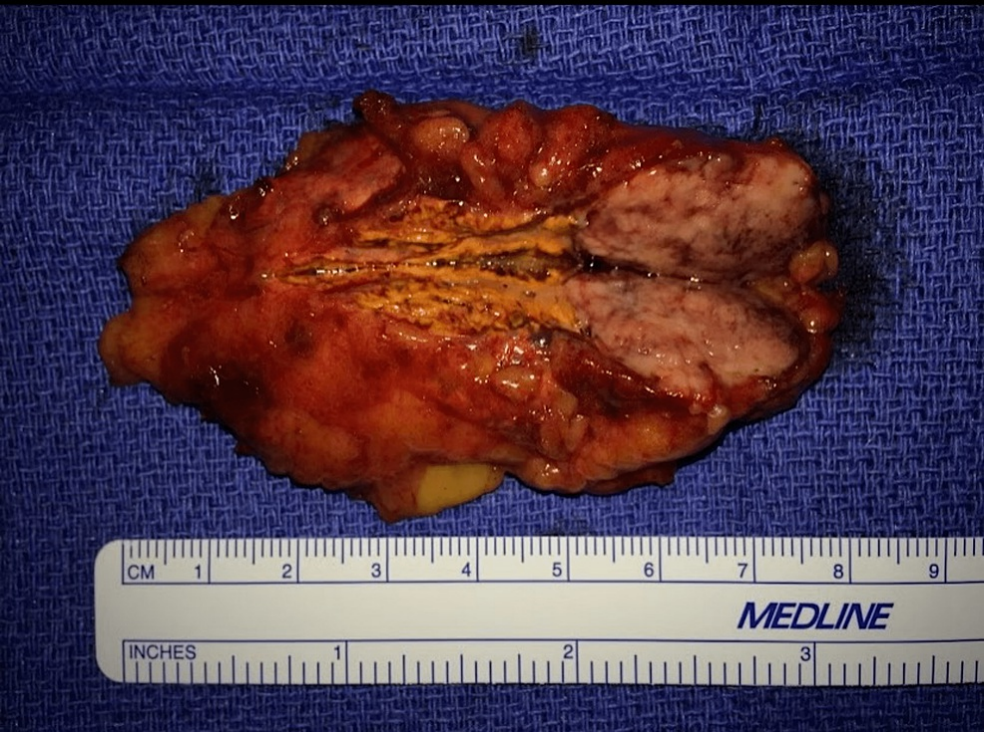
Pathology specimen of right adrenalectomy

A right laparoscopic adrenalectomy was performed with the removal of the right adrenal gland and 2.2 cm right adrenal nodule (Figure 4). Pathology of the mass confirmed a pheochromocytoma with negative margins. Her post-surgical clinical course did not have any complications, and she did not have any subsequent episodes of hypokalemia after her initial ER visit or reports to the ER with any symptomatic presentation of a pheochromocytoma.

## Discussion

Pheochromocytomas are rare tumors of the adrenal gland that arise from chromaffin cells and secrete catecholamines. Classically, pheochromocytomas present with symptoms, including headaches, profuse diaphoresis, and paroxysmal hypertension. A prospective study to estimate the incidence of pheochromocytomas in Spain from 1980 to 1992 found that the incidence during the period was 2.06 per a million [10]. Likewise, a systematic review of databases, including PubMed, Medline, and Embase, discovered that the mean incidence of pheochromocytomas from 1995 to 2015 was between 0.04 and 0.21 per 100,000 person-years with the mean age of diagnosis between 43 and 56 years old in the Netherlands [11]. Another study performed in Alberta, Canada, from 2012 to 2019 discovered an incidence of pheochromocytomas of 0.66 per 100,000 people per year [9]. Although pheochromocytomas are very rare, patients with adrenal incidentalomas should have proper testing.

Although we associate this tumor with the triad of headache, sweating, and tachycardia, there has been an increase in the diagnosis of pheochromocytomas in asymptomatic patients. The patients have unrelated symptoms and imaging leads to the discovery of an adrenal mass or incidentaloma. Studies done have shown a significant amount of adrenal incidentalomas being pheochromocytomas [3,5,9,12,13]. This increase in pheochromocytoma diagnoses may be due to factors, including increased diagnostic testing, advances in technology, and differences in geographic location. Because of the increase in adrenal incidentalomas being found as pheochromocytomas, all adrenal incidentalomas should be worked up by laboratory testing to look for hormone production. By testing for hormone production, the exact nature of an incidentaloma may be explained, and thus a possible pheochromocytoma could be found. Pheochromocytomas should be distinguished from other hormone-producing tumors, such as those seen in Cushing’s syndrome and Conn's syndrome. Cushing’s syndrome should be tested by either an overnight low-dose dexamethasone suppression test, late night salivary cortisol test, or 24-hour urinary free cortisol evaluation [14]. Aldosterone-producing tumors, such as those in Conn's syndrome, are tested for through increased measurement of morning plasma aldosterone and plasma renin activity [14]. Pheochromocytomas are tested for by checking for elevated 24-hour urinary fractionated metanephrines or elevated plasma fractionated metanephrines [3]. Nonetheless, it is important to recognize whether these adrenal masses are functional. Our patient was found to have an adrenal mass that was not associated with her nausea, and systematic testing determined it to be a pheochromocytoma. Her systematic testing did not show any evidence of other hormone-producing tumors, such as those seen in Cushing’s syndrome or Conn's syndrome. By distinguishing between different hormone-producing tumors, treatment and prevention of life-threatening situations could be made.

One concern with unrecognized pheochromocytomas is the possibility of a hypertensive crisis or hypertensive emergency. There may be associated metabolic derangements, coagulopathy, thromboembolic events, adrenal capsule rupture risk, hemodynamic instability, and end-organ dysfunction with a pheochromocytoma crisis [15]. Our patient was taking atenolol for her hypertension, which was concerning given that she had a silent pheochromocytoma. Beta-adrenergic blockade, such as through the use of atenolol, is contraindicated in patients with pheochromocytomas. Patients need to have alpha-adrenergic blockade first as beta-adrenergic blockade may cause a hypertensive or pheochromocytoma crisis due to unopposed alpha-adrenergic response. There have also been other instances of hypertensive crisis in patients with undiscovered pheochromocytomas due to various reasons. There was a reported case of a pregnant female who presented in a hypertensive crisis with headaches, tachycardia, and severe arterial hypertension of 220/120 mmHg that was triggered by metoclopramide and labetalol [16]. Further workup for the female revealed a right adrenal pheochromocytoma. Thus, this further stresses the importance of the recognition of pheochromocytomas in patients to prevent the occurrence of hypertensive crisis that may occur from medication use. In another instance, there was a 63-year-old male who suffered a hypertensive crisis during anesthetic induction during a catheter ablation leading to abortion of the procedure and was subsequently diagnosed with bilateral pheochromocytomas upon further workup [17]. One physician also found that 19 out of 27 patients he cared for had adrenal incidentalomas, with six having adrenal biopsies at outside hospitals due to failure to diagnose the incidentalomas correctly as a pheochromocytoma. Biopsies of pheochromocytomas could pose risks of catecholamine spillage leading to a hypertensive crisis [3]. Overall, this stresses the importance of diagnosing pheochromocytomas to prevent disastrous outcomes for individuals, such as our patient. In fact, many patients are diagnosed with pheochromocytomas only at autopsy [18]. Thus, it is important to work up patients with adrenal incidentalomas to find if any are pheochromocytomas. Our patient was advised not to attend her high school reunion due to the concern of a life-threatening hypertensive event. It is also possible that if she were to have any future procedures, a hypertensive crisis could have occurred if her pheochromocytoma was not discovered and removed.

The treatment of choice for pheochromocytomas is surgical excision of the tumor [1]. However, it is important to prevent a pheochromocytoma crisis both before and during intraoperative procedures, which is why our patient was given prazosin, an alpha-adrenergic antagonist, to prevent catecholamine binding onto alpha-receptors during any spillage. For these reasons, alpha-blockade is a standard management before surgery. Phenoxybenzamine is also another alpha-blockade medication used for the prevention of hypertensive crises in patients and could have been another option given to our patient. It is also recommended by the European Society of Endocrinology Clinical Practice Guidelines to follow up on patients for at least 10 years to screen for tumor recurrences or metastasis, such as screening for increased plasma or urinary metanephrines and normetanephrines annually [19]. The reason for this is that if the tumor recurs, it is important to monitor and prevent any dangerous hypertensive crises from happening. Thus, we recommend that our patient have follow-up screening to ensure that there is no reappearance of any pheochromocytomas and ensure her safety.

Our patient initially presented to the emergency room with nausea and found to have hypokalemia. There could be several reasons for this. It has been previously reported that increased levels of epinephrine may be related to episodes of hypokalemia, and there have been a few instances of patients with pheochromocytomas that have presented with hypokalemia [20,21]. Since epinephrine is a possible catecholamine that may be released from pheochromocytomas, the patient’s hypokalemic presentation at the ER could be due to this. Our patient’s pheochromocytoma could have released more epinephrine than within normal limits. Increased epinephrine seen in pheochromocytomas may cause intracellular shifting of potassium through beta-2-receptor stimulation [21]. Because of the possibility that our patient may have had increased epinephrine production from her pheochromocytoma, there could have been intracellular shifting of potassium causing her lower potassium serum levels. Unfortunately, she did not have her epinephrine levels checked during her ER visit. However, our patient had never had a hypokalemic episode again after her initial visit. At the ER, the hypokalemia was attributed to a common side effect of hydrochlorothiazide and that diuretic was discontinued [22]. Unfortunately, we will never know if her hypokalemia was due to her pheochromocytoma. Another hypothesized reason is that high plasma renin and aldosterone may occur in pheochromocytoma patients, which could cause hypokalemia [23]. Our patient had normal renin and aldosterone levels, which ruled this out as a cause of her low potassium levels. Again, it is crucial to work up adrenal incidentalomas for hormone production. More studies are needed to discern the relationship between potassium levels and pheochromocytomas. 

This case exemplifies the importance following up the results from the ER. The meticulous attention to detail with follow-up imaging led to the diagnosis of a silent pheochromocytoma. A potential limitation to this case is that we could have measured the patient’s catecholamine levels. It is difficult to attribute her hypokalemia to epinephrine release without having this value. Of course, hydrochlorothiazide is a well-known cause of hypokalemia. We also acknowledge that we have only presented one case of a silent pheochromocytoma as opposed to having a series of patients.

## Conclusions

We present a case of a patient to highlight an incidentally discovered silent pheochromocytoma. This case adds to the literature on the importance of following up on minimally abnormal labs found in the ER and reviews the essential workup for an adrenal incidentaloma. The minimally elevated creatinine led to a renal US that discovered a possible adrenal mass. Thereafter, an abdominal CT and MRI were done, and workup for the adrenal incidentaloma was begun. The pheochromocytoma was confirmed on biochemical labs, which revealed elevated metanephrines and normetanephrines, upon which she had a subsequent resection through a right adrenalectomy and pathologic confirmation of a pheochromocytoma. Our patient could have had a pheochromocytoma crisis at any time even though she was asymptomatic. Now, we recommend that the patient should have continued follow-up screening of plasma or urinary metanephrines and normetanephrines annually to monitor for recurrence or metastasis for at least 10 years after the removal of her pheochromocytoma. It is important for PCPs to carefully review any ER or discharge records from their patients and complete appropriate follow-up testing. Many times, no further testing is required. However, sometimes, you may make a rare diagnosis, such as a pheochromocytoma.
